# Safety Risk Assessment and Classification of Cadmium in Grain Processing Products

**DOI:** 10.3390/foods14111882

**Published:** 2025-05-26

**Authors:** Qingchuan Zhang, Wenjie Dou, Zheng Wang, Xuemei Xu, Tongqiang Jiang

**Affiliations:** 1 National Engineering Laboratory for Agri-Product Quality Traceability, Beijing Technology and Business University, Beijing 100048, China; zqc1982@126.com (Q.Z.); 2431022332@st.btbu.edu.cn (W.D.); 2 China International Electronic Commerce Center, Beijing 100083, China; vicky9710@163.com

**Keywords:** processed grain products, cadmium, risk assessment, risk classification, k-means++

## Abstract

As a staple food for people worldwide, processed grain products, including rice, wheat flour, etc., have been the major source of the toxic element cadmium for human exposure. Indeed, cadmium contamination in processed grain products directly affects food security and human health, especially in countries with a grain-based diet like China. By analyzing the cadmium levels in processed grain products across 20 provinces and cities in China during the period 2023–2024, we have developed an improved k-means++ algorithm that determines the optimal clustering number through a voting scheme. This algorithm has enabled us to establish a risk classification model, which provides an objective and rapid assessment of risk levels based on data-driven analysis. The results of the risk classification reveal that the risk levels of cadmium contamination in processed grain products can be classified into five levels, with high-risk products accounting for only 2.81% of the total. As for different types of processed grain products, the risk levels of rice and other processed grain products are higher than that of wheat. In addition, the risk levels of southern provinces/cities in China are higher than those of northern provinces/cities.

## 1. Introduction

Cadmium (Cd) is a primary type of contaminant in soil, primarily originating from industrial activities, agricultural practices (such as the use of phosphate fertilizers), and mining operations. It causes widespread concern because of its non-biodegradability and persistence in soil, and its ease of bioaccumulation in plant tissues [1]. Cd is much more readily absorbed via the roots of crop plants (e.g., grain) growing in Cd-contaminated soil than other heavy metals, and is subsequently deposited in the edible parts of plants; it is, therefore, the most talked about issue in the food chains [2]. Cd is recognized as a highly toxic element and classified as a Group I human carcinogen, posing substantial risks to human health [3]. Previous studies have found that Cd causes skeletal damage, reproductive effects, and cancers, along with long-term exposure to a variety of tissues, including the kidney and peripheral nervous systems [4,5].

Grain-derived products (e.g., rice, cereals) constitute the staple food for the majority of the world’s population. As the primary source of food yield, cereals contribute over 20% of the global dietary energy supply [6]. Grain processing products are an excellent source of carbohydrate, fueling vital physiological processes in humans, containing fiber that facilitates gastrointestinal function, and are characterized by minimal cholesterol and saturated fat content, making them beneficial for cardiovascular health [7]. In certain countries (e.g., Bangladesh and Cambodia) rice can constitute up to 70% of the dietary energy intake of their people [8]. Meanwhile, a major exposure route of Cd globally is wheat and rice consumption [9] and more than 70% of dietary intake of Cd is via the food chain [10]. Cd contamination in grain has become a serious food safety concern worldwide [1,11]. Food safety incidents caused by heavy metal hazards in processed grain products, particularly Cd in rice, occur from time to time in China [12], posing a serious threat to consumer health and social stability. A survey reports that over 19% of China’s agricultural soil has been contaminated by various pollutants [13]. In southern China alone, more than 1.3×10^5^ km^2^ of agricultural land is contaminated by Cd, affecting many agricultural products per year. Most of the arable land in this region is planted with rice, and the contaminated products are distributed [14]. Therefore, strengthening the risk assessment of Cd in grain processing products is of great practical significance to reduce the safety risks of processed grain products and protect the health of consumers. Specifically, more attention should be paid to contaminated rice and preventive measures need to be taken [15].

In recent years, studies on Cd contamination in processed grain products have been conducted in different countries, such as Bangladesh [16], Ecuador [17], India [2], etc. Rice is a staple food in China, leading to much higher consumption compared with other regions [18]. Given the relatively serious Cd pollution in some regions of China, the distribution and concentration of Cd in rice grains have been widely studied in China. Yang [19] assessed the pollution situation of Cd in wheat grains from the major wheat-cultivation areas of Baoji, a typical agricultural area in Shaanxi, and the dietary health risks caused by consuming wheat grains [20]. The results show that the mean grain concentration of Cd is 0.01 mg/kg, which is remarkably lower than the tolerance limits of the Chinese food hygiene standard (GB 2762-2017 [14]). In addition, there are studies of grain-growing areas with known industrial pollution sources [10], wastewater input fields, and typical farmland [21]. In these studies, risk assessment of heavy metals in grain processing products is conducted in certain areas without estimating the health risk of Cd from grain consumption. Meanwhile, most of these studies use small amounts of detection data. Although there are a few studies that perform assessments with larger amounts of nationwide data [22], they provide no results on smaller scales, e.g., urban areas. Given the demand to assess Cd contamination in grain processing products in China, we perform a municipal food safety risk assessment nationwide using national sampling detection data released by the State Administration for Market Regulation.

In terms of risk classification, various qualitative, semi-quantitative, and quantitative models have been developed [23]. Generally, the risk classification methods include statistical methods, e.g., Monte Carlo (MC) simulation [24] and the risk matrix method [25]. For statistical methods, MC simulation, disease burden estimation, and other statistical methods are used in the iResk model of FDA with mathematical functions. However, the accuracy of MC simulation depends on the number of simulations performed, making it complex and indigestible, and requiring large amounts of computer resources. When the number is small, the accuracy of MC simulation is low. For the risk matrix method, the UK Veterinary Residues Committee (VRC) proposes a classification model. VRC often uses the risk matrix to classify early warning risks of dairy products, thus obtaining reasonable suggestions. When the risk matrix is graded, the level boundaries are artificially set with strong subjectivity [26]. In contrast, the k-means clustering algorithm classifies samples based on sample similarity in a data-driven manner [27,28], effectively reducing the influence of subjective factors. In the traditional k-means algorithm, poor centroid initialization can lead to suboptimal clustering results. The k-means++ algorithm addresses this by using a more strategic initialization approach, improving both convergence and cluster accuracy. Additionally, the k-means++ clustering algorithm exhibits rapid computational capability and flexibility in adjusting the cluster number [29,30]. Given the fast-processing power and low complexity of the k-means++ algorithm, and the desire for objective risk grading in a data-driven way, we adopt the k-mean++ algorithm for Cd risk classification in processed grain products to efficiently derive objective grading results.

The sampling data of cadmium contamination in processed cereal products during 2023–2024 have about 500,000 copies, as the State Administration for Market Regulation reported. We analyze the pollution levels and concentrations of Cd in three processed grain products, i.e., rice, wheat flour, and other processed grain products, and conduct the risk assessment of Cd contamination in processed grain products by establishing a variety of dietary exposure assessment models.

Then, using the national sampling data of cadmium in processed food products and consumption data from 2023–2024, we conduct dietary exposure assessment to calculate the dietary intake of Cd for residents, and conduct municipal food safety risk assessments in China. In addition, we apply the k-means++ clustering algorithm to determine the risk level of cadmium contamination in food processing products, which provided a quantitative basis for decision making and regulatory prioritization based on sampling data and risk levels. This tool allows for the development of measures to address the factors contributing to varying risk levels, enhancing food safety management.

## 2. Materials and Methods

### 2.1. Data Source

In this study, we select Cd, which causes metal contamination in processed grain products, as the subject of study. The total number of samples in this study is 506,700, obtained from the National Food Safety Sampling Inspection Information System in 2023 and 2024. The system contains sampling data collected at a density of four batches per thousand people each year, covering more than 95% of provinces, municipalities, and counties across China. These samples come from 187 cities across 20 provinces, ensuring that the dataset is both diverse and highly representative. The processed grain products consist of three different types, including rice, wheat flour, and other processed grain products (processed grain products other than rice and wheat flour).

To build the subsequent risk assessment model, we collect population consumption data and relevant toxicological data to calculate assessment indicators. Population consumption data for processed food products in 20 provinces are obtained from the fifth China total diet study [31]

Moreover, we acquire related toxicology data from the Food and Agriculture Organization of the United Nations, the World Health Organization (WHO), and the United States EPA. The reference dose of Cd is 0.001 μg/(kg d) [32], and the national limit standard (GB 2762-2017) for Cd is 0.2 (mg/kg) [21].

### 2.2. Data Preprocessing

During the process of data collation, we integrate the key information and delete the useless information. In calculating the mean Cd concentration, detection results marked as “not detected” are assigned a Cd concentration of 0 mg/kg [33]. These results are displayed as zero in this study. With respect to results with an extra symbol, e.g., “<”, we delete the symbol but retain the value [34].

After preprocessing the data, we conduct descriptive statistical analysis using four key indicators: range, mean, standard deviation, and variation coefficient. Specifically, the range reflects the extent of cadmium concentration variation, calculated as the difference between the maximum and minimum values observed in the samples. The mean indicates the average cadmium concentration across all samples for each province and product type, offering a general overview of contamination levels. The standard deviation measures the degree of dispersion in cadmium concentrations, showing how much the values deviate from the mean. The variation coefficient, defined as the ratio of the standard deviation to the mean, indicates relative variability and enables comparison across different product types and regions.

### 2.3. Dietary Exposure Assessment Method

In order to systematically investigate the effects of Cd hazards in processed grain products on humans, we select the following safety indices covering health and environmental dimensions to classify the risk levels and constructed a risk classification assessment model. We assess human health risks using target cancer risks (TCRs) and target hazard quotients (THQs) for both carcinogenic and non-carcinogenic effects. Additionally, we employ the Nemerow integrated pollution index (NIPI) for environmental risk assessment, as it integrates detection data and quantifies the contamination risk of the studied metal in the region [35,36].

#### 2.3.1. Nemerow Integrated Pollution Index

The NIPI reflects food contamination characteristics and is used to evaluate heavy metal contamination in both water and rice. The NIPI $P_{c\left(i,j\right)}$ of heavy metal j in processed grain product i is given by:

(1) $$P_{c\left(i,j\right)}=\sqrt{\frac{P_{max\left(i,j\right)}^{2}+P_{avg\left(i,j\right)}^{2}}{2}}$$

where $P_{max\left(i,j\right)}$ is the maximum value of the heavy metal j pollution index in processed grain products, and $P_{avg\left(i,j\right)}$ is the average value.

The pollution index is expressed as:

(2) $$P_{i,j}=\frac{X_{i,j}}{S_{i,j}}$$

where $P_{i,j}$ and $X_{i,j}$ are the pollution index and detection value of heavy metal j in processed grain product i, respectively, and $S_{i,j}$ is the national limit standard for heavy metal j in grain processing product i.

#### 2.3.2. Target Cancer Risk

We use TCR [19], which reflects the possible type of carcinogenic risk, to assess the carcinogenic risk of Cd.

TCR is given by:

(3) $$TCR=\frac{EF\times ED\times CSF_{j}\times EDI_{j}^{50}}{AT_{C}}$$

where EF is the exposure frequency (365 days/year); ED is the exposure period (70 years in the current study); $CSF_{j}$ denotes the carcinogenic intensity index of heavy metal j (kg·d/mg); $AT_{C}$ is the duration of the carcinogenic effect (365 days/years*exposure period, assumed to be 70 years in this study).

The $EDI_{j}^{50}$ calculation is given by:

(4) $$EDI_{j}^{50}=\frac{FC_{j}\times X_{i,j}^{50}}{W}$$

where $FC_{j}$ is China’s per capita daily consumption of processed grain product i (kg/d); $X_{i,j}^{50}$ is the 50th quantile (mg/kg) of Cd j detection results in a certain city; W is the average body mass of the population (60 kg in this study) [23].

#### 2.3.3. Target Hazard Quotient

THQ [37] shows the non-carcinogenic risk, which is based on the contaminant exposure and reference dose. THQ is expressed as:

(5) $$THQ=\frac{EF\times ED\times EDI_{j}^{95}}{AT_{C}\times RfD_{j}}$$

where $RfD_{j}$ (reference dose) is the oral reference dose of heavy metal j (kg·d/mg); $EDI_{j}^{95}$ is calculated as:

(6) $$EDI_{j}^{95}=\frac{FC_{j}\times X_{i,j}^{95}}{W}$$

where $EDI_{j}^{95}$ is the 95th quantile (mg/kg) of heavy metal j detection results in a certain city.

### 2.4. Risk Classification Method

We consider the likelihood of exceedance, exposure, and hazard of food contaminants to quantify the risk factors of contaminants, and establish the food safety risk assessment and classification model through the above three indicators.

Scientifically and accurately determining the level of classification is one of the main issues in food safety risk classification. In this study, we use the clustering algorithm to assess the risk levels of food contaminants. It has obvious advantage in time complexity compared with the traditional methods.

The process of k-means++ [38] is as follows (Algorithm 1):
**Algorithm 1: k-means++ clustering**Input:  *X*: a data set containing *N* samplesOutput:  Set of Centroids ($\mu_{p}$)
1:   Choose an initial center $\mu_{1}$ uniformly at random from *X*
2:   Choose the next center $\mu_{p}$, selecting $\mu_{p}=x^{\prime }\in X$ with probability
          $\frac{Md{\left(x^{\prime }\right)}^{2}}{\sum_{i=1}^{N}Md{\left(x_{i}\right)}^{2}}$
     *//where* Md(x) *denotes the minimum distance between sample x to the previously computed centroid;* $x^{\prime }$ *is the centroid selected from the given dataset X// *3:   Repeat Step (2) until we have chosen a total of ‘K’ Centers
4:   Proceed as with the conventional k-means++ algorithm in [38]
5:   Return $\mu_{p}$


Obtaining a reasonable value of the risk classification number K is the key to ensuring a reasonable food classification result. Therefore, in this study, we choose the number of clusters by the silhouette coefficient (SC) [39], Dunn index (DI) [40], and Davies–Bouldin index (DBI) [41] based on a voting scheme to determine and assess the quality of the clustering separation and homogeneity.

### 2.5. Clustering Evaluation Method

#### 2.5.1. Silhouette Coefficient

The silhouette coefficient shows how close each data point is to other data points within a cluster and how well clusters are separated from one another. In other words, it works based on the distance between each point within and between the clusters. The optimal distance corresponds with the maximum silhouette value that reflects a better partition.

(7) $$sp\left(i\right)=\frac{b\left(i\right)-a(i)}{Max\left\{a\left(i\right),b(i)\right\}}$$

where $sp\left(i\right)$ is called the silhouette width of point; a(i) is the mean distance between the ith point and all the other points in the cluster $P_{i},(i=1,2,\ldots ,n)$; and $b\left(i\right)$ represents the smallest of these distances. Thus, the silhouette value is between 1 and −1, where a high value indicates better clustering accuracy.

Finally, the silhouette index takes the average of sp(i) for all the samples to evaluate the clustering result as follows:

(8) $$SC=\frac{1}{N}\sum_{i=1}^{N}sp(i)$$

#### 2.5.2. Dunn Index

The Dunn index is an indicator that denotes the minimum value of the distance between clusters and the maximum value of the distance between elements in a cluster. In other words, the longer the distance between the clusters and the smaller the clusters, the better the clustering. In this case, the Dunn index will become larger with better clustering. The index definition is

(9) $$DI=\frac{\underset{i\neq j}{\min}\left\{d_{c}(C_{i},C_{j})\right\}}{\underset{1\leq l\leq k}{\max}\left\{∆(C_{l})\right\}}$$

where $d_{c}\left(C_{i},C_{j}\right)=\underset{x\in c_{i},y\in c_{j}}{\min}\left\{d(x,y)\right\}\;$or the distance between two clusters, $∆\left(C_{l}\right)=\underset{x,y\in c_{l}}{\max}\left\{d(x,y)\right\}$ or the diameter of $C_{l}$, d(x,y) is the Euclidean distance between two data elements, and is the number of clusters. Clearly, $DI\in \left[0,\infty \right]$ with larger values of DI indicates better clustering.

And compact clusters that are well separated in the feature space manifest themselves in small values of $\underset{i\neq j}{\min}\left\{d_{c}(C_{i},C_{j})\right\}$ and large values of $\underset{1\leq l\leq k}{\max}\left\{∆(C_{l})\right\}$, leading to a small value of DI.

#### 2.5.3. Davies–Bouldin Index

The Davies–Bouldin index is a function of the ratio of the sum of the distance between two signals within the same cluster to the separation between different clusters. A lower value indicates a better separation between clusters and better cohesion within clusters. Mathematically, the above can be presented as follows:

(10) $$avg\left(C_{i}\right)=\frac{2}{k(k-1)}\sum_{1\leq i<j\leq k}D_{ij}$$

(11) $$d_{cen}\left(C_{i},C_{j}\right)=D_{\left(\bar{x},\bar{y},\bar{z}\right)}c_{i}(\bar{x},\bar{y},\bar{z})c_{j}$$

(12) $$DBI=\frac{1}{N}\sum_{i=1}^{N}\max\left(\frac{avg\left(C_{i}\right)+avg\left(C_{j}\right)}{d_{cen}\left(C_{i},C_{j}\right)}\right),(j\neq i)$$

where Equation (10) represents the average Euclidean distance of k points within $C_{i}$; Equation (11) is used to calculate the Euclidean distance between centers $C_{i}$ and $C_{j}$; and Equation (12) gives the DBI value of current cluster results with *N* clusters.

#### 2.5.4. Voting Scheme

After calculating three clustering evaluations, we use a simple voting strategy to increase the reliability of the evaluation method. On the basis of three cluster evaluation indices, the numerical performance of each number of clusters is obtained in the following scheme, where A is the maximum number of clusters:

(i) The number of clusters with the best index performance is given A points;
(ii) The number of clusters with the second-best performance is given (A-1) points;
(iii) The number of clusters with the third-best performance is given (A-2) points;
(iv) The number of clusters with the fourth-best performance is given (A-3) points;
(v) The number of clusters with the worst performance is given 1 point.

Following that, the points of each cluster evaluation index are accumulated as a function of the number of clusters and the number of clusters with the global maximum in points is thus determined and chosen for the current evaluation indices combination.

With the k-means++ clustering algorithm, we can select the optimal parameter and cluster number combination. The final clustering results can provide a scientific and reasonable assessment of the risk level of cadmium in processed grain products, and further enhance the food safety control.

## 3. Results

### 3.1. Cadmium Pollution in Grain Processing Products

The Cd concentrations in rice, wheat flour, and other processed grain products collected from the National Food Safety Sampling Inspection Information System have been statistically analyzed as range, mean, standard deviation, and variation coefficient, yielding the pollution situation of Cd in China during 2023–2024.

**Figure 1:**
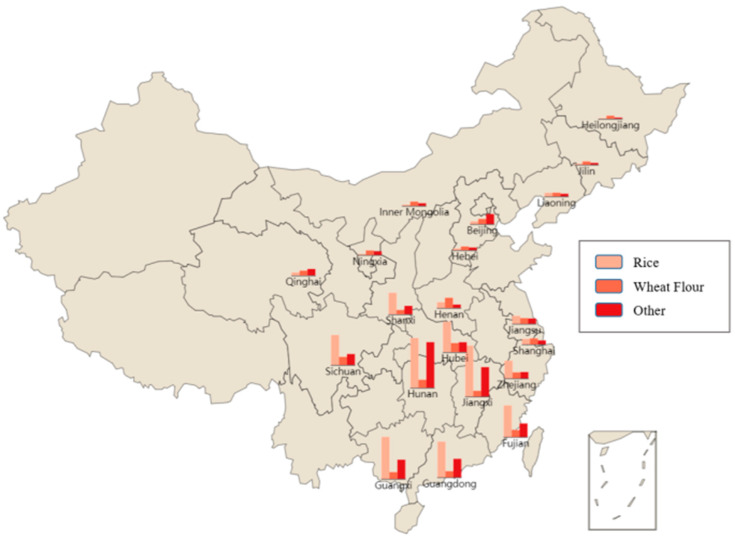
Spatial distribution of Cd concentration in China during 2023–2024.

Table 1 shows the national differences in the mean Cd concentrations in three types of processed grain products. As shown in Table 1, wheat flour contains less Cd than rice and other processed grain products, which have a mean content of 0.01369 mg/kg. Rice has a higher average Cd content than wheat and the other processed grain products, reaching 0.04887 mg/kg; however, the coefficient of variation of the other processed grain products (2.75427) is greater than that of rice (1.39111), indicating that the degree of dispersion is greater for the other processed grain products. Then, the spatial distribution of Cd concentration is visualized in Figure 1.

According to Table 1 and Figure 1, rice, wheat flour, and other processed grain products with high Cd are mainly concentrated in the central and south of China, and the highest concentrations of those three products are 0.01273 mg/kg (rice), 0.02267 mg/kg (wheat flour), and 0.10089 mg/kg (other) in Jiangxi, Henan, and Hunan provinces, respectively.

### 3.2. Risk Assessment Results

#### 3.2.1. Provincial Risk Assessment Results

Cd exposure typically occurs through food, with factors such as intake rate, frequency, metal concentrations, exposure duration, and body weight influencing the associated health risks from rice consumption. Therefore, in addition to statistically analyzing Cd concentration, health assessment is also critical for a scientific and comprehensive investigation of the Cd hazard. Meanwhile, many other international studies have estimated Cd concentrations and used them in risk assessment calculations, as shown in Table 2.

In addition, to investigate the spatial distribution of the health assessment indices for Cd in processed grain products in 20 Chinese provinces/cities over the period 2023–2024, we depict Table 2 in a heat map.

**Figure 2:**
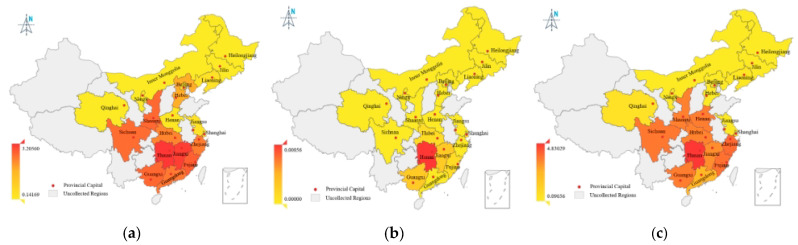

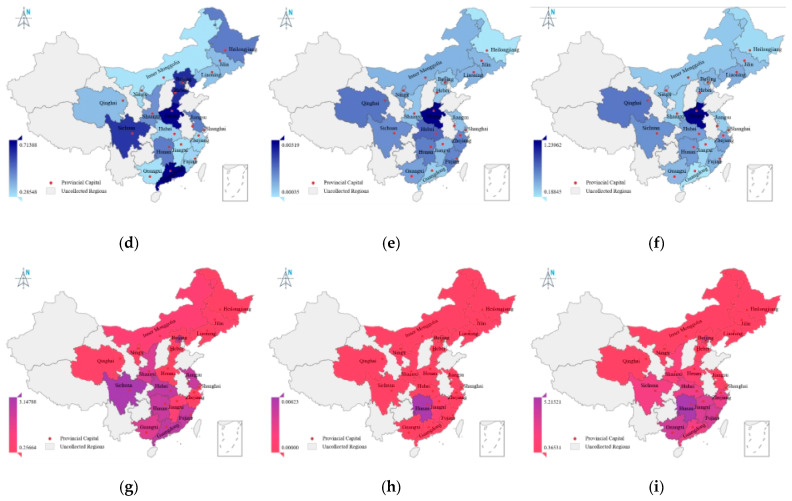
Spatial distribution of health assessment indices for Cd in grain processing products in 20 Chinese provinces and cities over the period 2023–2024. (**a**) Spatial distribution of NIPI in rice; (**b**) Spatial distribution of TCR in rice; (**c**) Spatial distribution of THQ in rice; (**d**) Spatial distribution of NIPI in wheat flour; (**e**) Spatial distribution of TCR in wheat flour; (**f**) Spatial distribution of THQ in wheat flour; (**g**) Spatial distribution of NIPI in other processing product; (**h**) Spatial distribution of TCR in other processing product; (**i**) Spatial distribution of THQ in other processing product.

It can be seen from Table 2 and Figure 2 that, for NIPI, rice (3.2056, 3.03147) in Hunan province and Jiangxi province and other processed grain products (3.14788) in Sichuan province located in south of China are relatively high (see Figure 2a,g), whereas NIPI in wheat flour are relatively low and the highest value is in Guangdong province (0.71388), while some central provinces such as Henan and Hebei also have higher NIPI values (see Figure 2d). Regarding TCR, in rice, eight provinces have TCR values above 10^−4^, and all of them are located in southern China, with the highest value of 0.00856 in Hunan province 268 (see Figure 2b); in wheat flour, half of the provinces have TCR values above 10^−4^, and only Henan and Liaoning provinces are located in northern China, with the highest value of 0.00319 in Henan (See Figure 2e). Similarly, in other processed grains, the provinces with TCR values above 10^−4^ are Hunan (0.00623), Jiangxi (0.00428), and Guangxi (0.00157) located in the south of China and Qinghai (0.00120) located in the north of China (See Figure 2h). As for THQ, half of the provinces have a THQ value above 1 in rice, and most of these provinces are in the south of China (See Figure 2c), with the exception of Henan and Shaanxi provinces, and the highest value is in Hunan province (4.83029), followed by Zhejiang province (2.93829). Similarly, for the other processing grain products, nearly half of the provinces have a THQ value above 1 and all of them, with the exception of Beijing, are in the south of China (See Figure 2i). The highest value is also found in Hunan province, at 5.21521, followed by Guangxi province (2.20096) and Fujian province (2.00143). Regarding wheat flour, however, only wheat flour from Henan province has a THQ value above 1, at 1.23962, while the rest of the provinces are less than 1 (See Figure 2f).

#### 3.2.2. Municipal Risk Assessment Results

**Figure 3:**
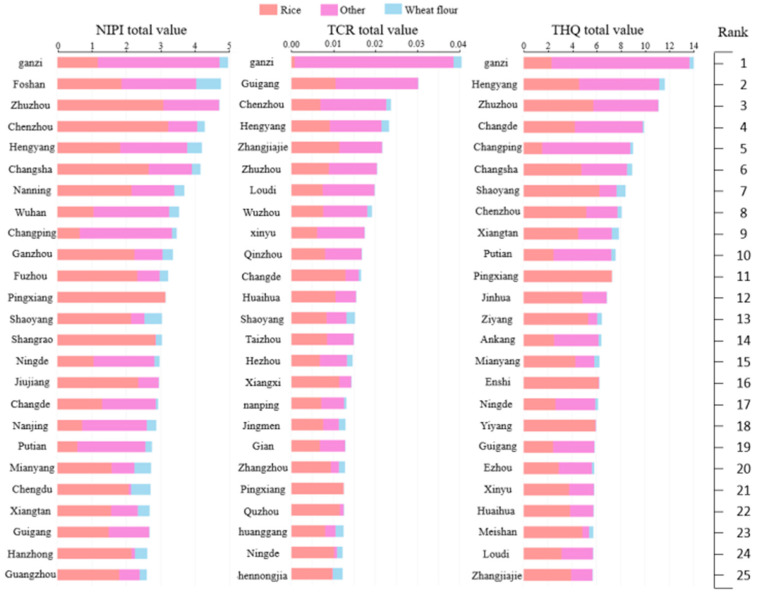
Municipal health assessment indices—NIPI, TCR, THQ—for Cd in grain processing products in China during 2023–2024.

In order to provide a more comprehensive and specific analysis of Cd contamination of grain processing products in China, we also perform a municipal health risk assessment for China during 2023–2024, and the results of the dietary assessment for three processed grain products are presented in Figure 3.

We calculate the total NIPI, TCR, and THQ for these products across various cities, with part of the results presented in the figure. As seen in Figure 3, Ganzi City in Sichuan Province has the highest total NIPI, TCR, and THQ values, indicating a significant health risk due to Cd contamination. Other cities with high-risk values, such as Chenzhou, Zhuzhou, and Hengyang, are primarily located in southern provinces like Jiangxi, Hunan, and Sichuan. These areas show elevated contamination levels, largely due to higher Cd concentrations in rice and other grain products compared with wheat flour.

Cities such as Ganzi and Chenzhou exhibit particularly high-risk values, primarily due to agricultural practices such as the use of contaminated water sources and soil management techniques that increase the uptake of cadmium by rice plants. The high consumption of rice in these regions further contributes to increased dietary cadmium intake, resulting in higher overall contamination risk. In particular, the combination of agricultural practices that enhance cadmium accumulation in rice and the high dietary reliance on rice leads to an elevated health risk in these areas, which is reflected in the high NIPI, TCR, and THQ values.

### 3.3. Risk Classification

#### 3.3.1. Determination of Clustering Center

**Figure 4:**
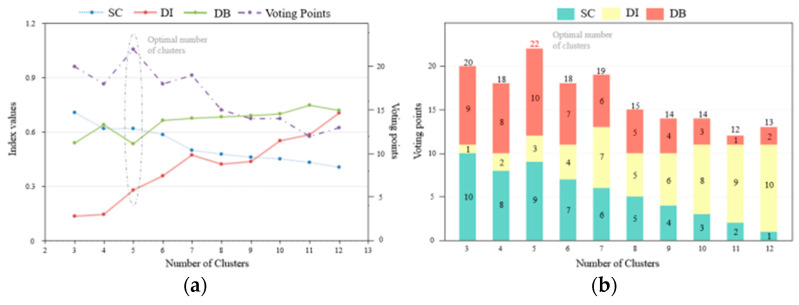
(**a**) Evaluation of three cluster index values; (**b**) evaluation of three cluster index values voting points of k-means++ clustering. The optimal number of clusters is 5.

After comprehensively assessing the Cd hazard in processed grain products, we perform spatial analysis of Cd distribution in China. In order to make the automatic determination of the optimal number of clusters robust, we combine the calculation of the clustering evaluation indices combined with the voting scheme and show the results in Figure 4.

Figure 4a shows three indices, namely the silhouette coefficient (blue), Dunn index (red), and Davies–Bouldin (green), and the voting points (purple). Figure 4b plots the points for each index and the final voting points. The final optimal number of clusters is 5, with 22 points. It is noted that the optimal number of clusters manifests itself in a small value of DB, while it corresponds to large values of the SC and DI index.

#### 3.3.2. Risk Level of Cadmium

**Figure 5:**
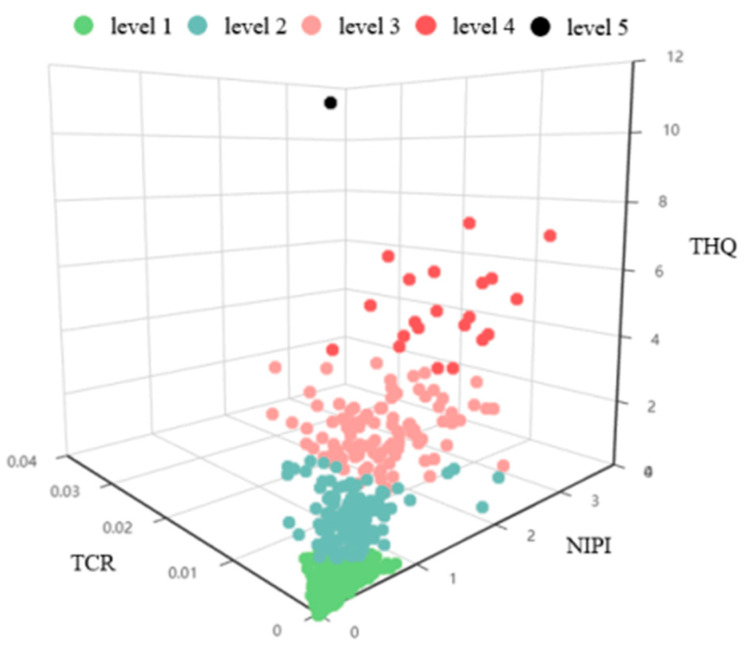
Risk classification results of Cd contamination in grain processing products.

Figure 5 depicts the risk classification of Cd contamination in processed grain products using the k-means++ clustering method, adopting the optimal clustering number, for three different food products.

As shown in Figure 5, the Cd contamination of processed grain products in cities with risk levels 1 and 2 accounts for 80.33% of the total, and that with levels 4 and 5 (high-risk levels) accounts for only 2.81%. Only other processed grain products in Ganzi City, Sichuan Province were level 5. However, the classification results in this analysis reflect the relative risk levels of Cd contamination in processed grain products, rather than the absolute risk. The higher the risk level, the greater the priority for attention. Table 3 shows the combination of food products and cities with high-risk levels.

As can be seen from Table 3, rice and other processed grains account for 72.72% and 27.27% of high risk of Cd contamination, respectively, while wheat flour does not appear among the high risk groups; the provinces with the most high-risk cities are Hunan and Sichuan, accounting for 50% and 22.72%, respectively. With the exception of Changping in Beijing and Hengshui in Hebei, the high-risk cities are all located in southern China.

## 4. Discussion

In order to assess the health risks of Cd in three types of processed grain products, we perform statistical analysis of Cd concentrations. The results show that the mean Cd concentrations (see Table 2) in rice, wheat flour, and other processed grain products in China are far below the maximum allowable limit of 0.2 mg/kg (see Table 4). While Cd pollution is frequently detected, the concentrations remain within a safe range (<0.2 mg/kg). Compared with a 2010 study [18], the average Cd concentration has decreased from 0.05 mg/kg to 0.02 mg/kg, suggesting that the risk of cadmium contamination has been notably reduced and is now largely under effective control nationwide. These reductions in Cd contamination can largely be attributed to the implementation of China’s effective pollution control measures. The government has introduced stricter regulations on heavy metal emissions, enforced environmental protection laws more rigorously, and initiated targeted agricultural pollution management policies, including soil remediation projects and enhanced monitoring of food safety standards. Furthermore, the total mean Cd concentration in rice (0.04887 mg/kg) during 2023–2024 was almost the same as the previous studies in Bangladesh (0.04400 mg/kg) [16]. Compared with wheat flour (0.01369 mg/kg), the mean pollution concentration of Cd in rice exceeds it by more than three times, almost consistent with the results in previous research in Jiangsu province [37], suggesting that rice in certain regions may be affected by Cd contamination originating from production areas or along the supply chain. Meanwhile, generally, the concentration of Cd is higher in the southern regions than in the northern regions; similarly, it is higher in the central south regions (e.g., Hunan) than in the other southern regions, and it is higher in the southwest regions than in the south eastern coastal areas (see Figure 1 for the details), which might result from the consumption of rice among residents of southern, central south, and southwest regions being more than those of other regions and consistent with the results of Wei et al. [21].

Health risk assessment from toxic metals is crucial as it provides valuable information on risk management and enables action required to minimize risk so that human health is protected, thus a comprehensive risk assessment of Cd was performed. We first performed the health risk assessment of Cd in 20 provinces in China during 2019 and 2020, and the spatial distribution suggested that, overall, the risk assessment indices—NIPI, TCR, THQ—for Cd were higher in the south than in the north of China, a trend that was consistent with the results of Cd pollution (see Figure 1). In addition, central China (e.g., wheat in Henan) and south-central China (e.g., rice in Hunan and Jiangxi) had higher assessment indices (see Figure 2d). Meanwhile, the health risk assessment indices for Cd were higher in the southeastern coastal areas compared with the inland provinces (see Figure 2g,i). The above phenomenon may be due to the different dietary structures among the residents in different regions. The intake of Cd in the south and center of China (e.g., Jiangxi, Hunan, Henan) from processed grain products was higher than in other regions, which is consistence with the conclusion of the fourth and fifth China total diet study (TDS) and other previous research. Then, in municipal heath risk assessments, the southern cities of Ganzi, Foshan, Zhuzhou, Chenzhou, and Hengyang, which are mainly located in the southern provinces of Hunan and Sichuan in China, have a high total risk assessment (see Figure 3). Our investigation is consistent with the findings that Cd pollution is more severe in Hunan, Guangdong, and Sichuan provinces.

Moreover, we use a k-means++ clustering algorithm to realize the risk classification of Cd contamination in processed grain products. The optimal clustering number 5 is determined by three clustering evaluation indices integrating the voting scheme. Since cluster validity indices are often affected differently by outliers and dataset structures, strengths of different indices are effectively combined by the voting strategy, while weaknesses are reduced. And an improved automated determination of the optimal number of clusters can be obtained through the combined evaluation of multiple indices (see Figure 3). As Sause et al. pointed out, the cluster evaluation indices used in this study were chosen because of their low numerical complexity. Alternatively, there are a variety of other cluster separation methods available, but they usually come at a higher cost in terms of computation. As a result, they are less desirable for automatically screening a large number of feature combinations.

In contrast with the traditional risk classification method of dietary exposure assessment, the risk classification model allows us to consider the combined effects of three indices (see Figure 5) in a comprehensive and objective manner. Regarding the three indicators in our model, we use THQ to characterize the risk of non-carcinogenic dietary intake for dietary exposure assessment, and TCR to characterize the risk of chronic dietary intake, combined with the NIPI to consider the need for a comprehensive evaluation of risk management to some extent. However, the classification results just show the relative risk of health hazards, and do not reflect the absolute risk. Therefore, the combinations with risk levels 3, 4, and 5 mainly indicate the priority of concern.

The risk classification results suggest that cities with higher risk levels are concentrated in Hunan province (e.g., Zhuzhou and Hengyang) and Sichuan province (e.g., Ganzi) (see Figure 5 and Table 3), and rice and other processed grain products, compared with wheat flour, account for greater high risk levels, which is consistent with the results shown in Figure 1 and Figure 3. For cities in Hunan province, mining activities are the main anthropogenic pollution source of Cd. In addition, the legacies of excavation operations, transport, and selective smelting activities within Hunan have resulted in the generation of large quantities of mine waste. The high content of Cd in the mining waste, especially the tailings, may have caused the grain processing products in Hunan province to have high risk levels. Additionally, Hunan exhibits high levels of waste water, industrial residue, and waste gas, significantly increasing the presence of heavy metals in the environment and potentially causing contamination of processed grain products. For cities in Sichuan province, Xu et al. found that the exploitation activities in the Maoniuping REE mining area (Sichuan province) have had direct and indirect impacts on Cd accumulation increases in the floodplains downriver since1986. Some research also discuss the impacts of social and economic development of Cd on farmland in Chengdu city of Sichuan province, including increasing industrial enterprises, excessive fertilizer application, and development of the transport industry. The combination of these causes may have contributed to the high risk of Cd contamination of processed grain products in Sichuan province [27].

The food safety supervision and sampling process in our country is continuous, constituting a routine and long-term monitoring task. The national supervision and sampling system continuously aggregates the detected contaminant concentrations, and more heavy metal contamination data are expected to be collected in the future.

## 5. Conclusions

In this paper, we statistically analyze the concentrations of Cd in processed grain products. The results suggest that mean Cd concentrations in various processed grain products are all within the limit standards. To further analyze the health risks of Cd, we construct a dietary exposure assessment model.

The results show that the risk assessment indices for Cd are higher in the south than in the north of China, particularly in Hunan, Jiangxi, and Sichuan provinces. Moreover, regarding municipal levels, the southern cities have high total risk assessments. Then, on the basis of exposure assessment, the risk classification model established in this study uses the k-means++ clustering algorithm to conduct an objective risk level assessment in a data-driven manner. The results show that high risk accounts for only 2.81% of the total. Only processed grain products in Ganzi city, Sichuan province, are at level 5, indicating that priority of attention needs to be given. Moreover, we mainly conclude two differences derived from the exposure assessment and risk classification models, i.e., the levels of risk are higher for rice and other processed grains than for wheat, and the levels of risk are higher in the southern provinces/cities of China than in the northern provinces/cities. Therefore, the risk of Cd to the southern population, especially rice and other processed grain products, is of concern. Future studies should focus on enhancing the risk classification assessment model by incorporating indices that integrate and quantify the impacts of heavy metals on specific target organs. Moreover, given the continuous and large-scale nature of the national food safety supervision system, researchers can leverage the growing dataset to perform more detailed and dynamic risk assessments. Not only that, expanding the range of heavy metals analyzed will further improve the comprehensiveness of food safety evaluations across diverse regions and food categories.

## Figures and Tables

**Table 1:** Statistics characteristics of Cd for 20 provinces in China during 2023–2024.

Province	Products	Range (mg/kg)	Mean (mg/kg)	Standard Deviation	Variation Coefficient
Shanghai	Rice	0.16000	0.01260	0.02154	1.70917
	Wheat flour	0.03800	0.01363	0.00747	0.54822
	Other	0.07200	0.00923	0.01430	1.54953
Inner Mongolia	Rice	0.11000	0.00291	0.00910	3.12391
	Wheat flour	0.04000	0.00975	0.00597	0.61213
	Other	0.23000	0.00621	0.01315	2.11749
Beijing	Rice	0.18000	0.00662	0.01954	2.95102
	Wheat flour	0.08200	0.01207	0.01003	0.83087
	Other	0.75000	0.02294	0.08647	3.76889
Jilin	Rice	0.09800	0.00264	0.00652	2.46670
	Wheat flour	0.05100	0.00756	0.00718	0.95025
	Other	0.18000	0.00441	0.01315	2.98026
Sichuan	Rice	0.58000	0.06715	0.08546	1.27270
	Wheat flour	0.08400	0.01887	0.01347	0.71379
	Other	0.89000	0.02519	0.08833	3.50729
Ningxia	Rice	0.04000	0.00249	0.00497	1.99629
	Wheat flour	0.04200	0.01061	0.00810	0.76323
	Other	0.14000	0.00867	0.01898	2.18872
Guangdong	Rice	0.52000	0.07912	0.05992	0.75728
	Wheat flour	0.10000	0.01387	0.00959	0.69141
	Other	0.60800	0.04118	0.06208	1.50750
Guangxi	Rice	0.60000	0.09311	0.06649	0.71413
	Wheat flour	0.03900	0.01527	0.00858	0.56205
	Other	0.35000	0.04268	0.06159	1.44314
Jiangsu	Rice	0.20000	0.01766	0.02129	1.20557
	Wheat flour	0.06600	0.01261	0.01013	0.80349
	Other	0.53000	0.01187	0.04428	3.73079
Jiangxi	Rice	0.85000	0.11262	0.07652	0.67942
	Wheat flour	0.04000	0.01273	0.00747	0.58670
	Other	0.23000	0.06516	0.05304	0.81390
Hebei	Rice	0.33000	0.00432	0.01975	4.56663
	Wheat flour	0.08800	0.00874	0.01034	1.18327
	Other	0.12000	0.00644	0.01194	1.85387
Henan	Rice	0.17000	0.01282	0.03040	2.37192
	Wheat flour	0.09600	0.02267	0.01441	0.63551
	Other	0.07500	0.00768	0.01188	1.54634
Zhejiang	Rice	0.51000	0.03934	0.05523	1.40402
	Wheat flour	0.04200	0.01363	0.00810	0.59409
	Other	0.15000	0.01441	0.02382	1.65280
Hubei	Rice	0.47000	0.06689	0.05038	0.75318
	Wheat flour	0.04200	0.01981	0.00818	0.41272
	Other	0.62000	0.02171	0.05397	2.48594
Hunan	Rice	0.90000	0.10986	0.10554	0.96069
	Wheat flour	0.06600	0.01766	0.01058	0.59878
	Other	0.52000	0.10089	0.11111	1.10139
Fujian	Rice	0.65000	0.07029	0.07143	1.01620
	Wheat flour	0.04200	0.01616	0.00609	0.37670
	Other	0.55000	0.03059	0.06568	2.14671
Liaoning	Rice	0.20000	0.00836	0.01732	2.07111
	Wheat flour	0.04900	0.00867	0.00891	1.02725
	Other	0.15000	0.00638	0.01165	1.82807
Shaanxi	Rice	0.60000	0.04752	0.06668	1.40316
	Wheat flour	0.06200	0.00967	0.00875	0.90467
	Other	0.38000	0.01864	0.03265	1.75161
Qinghai	Rice	0.07100	0.00648	0.01008	1.55508
	Wheat flour	0.05000	0.01045	0.00830	0.79397
	Other	0.13000	0.01424	0.02562	1.79932
Heilongjiang	Rice	0.08200	0.00141	0.00522	3.68673
	Wheat flour	0.06800	0.00656	0.00823	1.25482
	Other	0.09500	0.00252	0.00887	3.52440
**Average**	Rice	0.90000	0.04887	0.06798	1.39111
	Wheat flour	0.10000	0.01369	0.01161	0.84851
	other	0.89000	0.01590	0.04380	2.75427

**Table 2:** NIPI, TCR, and THQ risk assessment indices of three grain processing products for 20 provinces in China during 2023–2024.

Province	Rice			Wheat Flour			Other		
	NIPI	TCR	THQ	NIPI	TCR	THQ	NIPI	TCR	THQ
Shanghai	0.56744	0.00030	0.46869	0.28548	0.00077	0.24568	0.25664	0.00031	0.44365
Inner Mongolia	0.38904	0.00000	0.20768	0.29112	0.00099	0.32882	0.81347	0.00041	0.39805
Beijing	0.63683	0.00000	0.40297	0.58608	0.00095	0.34383	2.65289	0.00000	1.37258
Jilin	0.34661	0.00000	0.26022	0.36456	0.00093	0.36031	0.63659	0.00000	0.36531
Sichuan	2.06431	0.00279	2.55259	0.60878	0.00144	0.57769	3.14788	0.00065	1.01163
Ningxia	0.14169	0.00000	0.15369	0.30632	0.00105	0.38423	0.49592	0.00042	0.45440
Guangdong	1.85964	0.00354	1.23811	0.71388	0.00063	0.18845	2.15453	0.00091	1.05923
Guangxi	2.14671	0.00675	2.42424	0.29616	0.00121	0.39553	1.24660	0.00157	2.20096
Jiangsu	0.70986	0.00072	0.56892	0.47514	0.00078	0.29998	1.87430	0.00011	0.38687
Jiangxi	3.03147	0.00674	2.03157	0.29682	0.00088	0.26731	0.84518	0.00428	1.81238
Hebei	1.16683	0.00000	0.35552	0.62532	0.00075	0.40542	0.42487	0.00000	0.38983
Henan	0.60275	0.00000	2.42990	0.6975	0.00319	1.23962	0.26655	0.00061	0.78425
Zhejiang	1.80848	0.00177	2.93829	0.31224	0.00154	0.49378	0.53277	0.00090	1.20911
Hubei	1.67844	0.00568	2.29040	0.32836	0.00192	0.51687	2.19337	0.00078	1.16200
Hunan	3.20560	0.00856	4.83029	0.48312	0.00162	0.52680	1.87276	0.00623	5.21521
Fujian	2.31150	0.00570	2.76165	0.31822	0.00155	0.41425	1.94755	0.00083	2.00143
Liaoning	0.70772	0.00045	0.64105	0.35186	0.00100	0.45251	0.53081	0.00040	0.37520
Shaanxi	2.12796	0.00082	2.35158	0.44370	0.00077	0.30048	1.34512	0.00082	0.91450
Qinghai	0.25207	0.00053	0.72869	0.36120	0.00176	0.70067	0.46237	0.00120	0.44365
Helongjiang	0.28996	0.00000	0.09656	0.48306	0.00035	0.24141	0.33599	0.00000	0.39805

**Table 3:** The combination of food products and cities at a high-risk level.

Grain Processing Products Category	City	Risk Level	Province
Other	Ganzi	5	Sichuan
Other	Changping	4	Beijing
Rice	Chengdu	4	Sichuan
Rice	Meishan	4	Sichuan
Rice	Mianyang	4	Sichuan
Rice	Ziyang	4	Sichuan
Rice	Pingxiang	4	Jiangxi
Rice	Hengshui	4	Hebei
Rice	Jinhua	4	Zhejiang
Rice	Enshi	4	Hubei
Other	Changde	4	Hunan
Rice	Changde	4	Hunan
Other	Zhuzhou	4	Hunan
Rice	Zhuzhou	4	Hunan
Rice	Xiangtan	4	Hunan
Rice	Yiyang	4	Hunan
Other	Hengyang	4	Hunan
Rice	Hengyang	4	Hunan
Rice	Shaoyang	4	Hunan
Rice	Chenzhou	4	Hunan
Rice	Changsha	4	Hunan
Other	Putian	4	Fujian

**Table 4:** Limit of Cd in this study.

	Rice	Wheat Flour	Other
Cd	0.2	0.1	0.2

Note: data source GB 2762-2017 [14]; for other grain processing products, we use 0.2 mg/kg as the maximum limit standard.

## Data Availability

The data presented in this study are available on request from the corresponding author. The data are not publicly available due to the data being available with the permission of the State Administration for Market Regulation Statistics.
